# Anticholangiocarcinoma activity and toxicity of the *Kaempferia galanga* Linn. Rhizome ethanolic extract

**DOI:** 10.1186/s12906-017-1713-4

**Published:** 2017-04-12

**Authors:** Asmare Amuamuta, Tullayakorn Plengsuriyakarn, Kesara Na-Bangchang

**Affiliations:** Center of Excellence in Pharmacology and Molecular Biology of Malaria and Cholangiocarcinoma, Chulabhorn International College of Medicine, Thammasat University, Phahonyothin Road, Klonglung District, Pathum Thani, 12120 Thailand

**Keywords:** Cholangiocarcinoma, *Kaempferia galangal* Linn, Ethyl-p-methoxycinnamate (EPMC), Cytoxicity, CL-6, Anti-CCA, Nude mouse xenograft model

## Abstract

**Background:**

Cholangiocarcinoma (CCA) is an important public health problem in several tropical and subtropical parts of the world particularly Thailand. Chemotherapy of CCA is largely ineffective and discovery and development of effective alternative drugs is urgently needed. The objective of the study was to confirm the anti-CCA potential as well as toxicity of the crude extract of *Kaempferia galangal* Linn. (rhizome) both in vitro and in animal models.

**Methods:**

The ethanolic extract of *K. galanga* Linn. rhizome, ethyl-p-methoxycinnamate (EPMC) and 5-fluorouracil (5-FU) were evaluated for their cytotoxic activities against CCA cell line (CL-6) using MTT cell proliferation assay. Acute and subacute toxicity of the extract were evaluated in ICR (Imprinting Control Region) mice according to the OECD (International Organization for Economic Co-operation and Development) Guideline. Anti-CCA activity was evaluated in CCA- xenografted nude mice.

**Results:**

Results of cytotoxicity test showed moderate activity of the extract and EPMC with median (95% confidence interval: 95% CI) 50% inhibitory concentration (IC_50_) of 64.2 (57.76–72.11) and 49.19 (48.16–52.29) μg/ml, respectively. The IC_50_ of 5-FU was 107.1 (103.53–109.64) μg/ml. The selectivity index (SI) values for the extract, EPMC and 5-FU against human normal cell line (OUMS) and cancer cell line (CL-6) were 2.2, 2.09 and 1.31, respectively. Toxicity testing revealed no overt toxic effect up to the maximum single oral dose of 5000 mg/kg body weight and up to daily dose of 1000 mg/kg body weight for 30 days. The extract at the maximum tolerated dose level of 1000 mg/kg body weight for 30 days exhibited promising anti-CCA activity in CL6-xenografted nude mice as determined by inhibitory activity on tumor growth (58.41%) and lung metastasis (33.3%), as well as prolongation of survival time (62 days).

**Conclusion:**

The *K. galangal* Linn. rhizome extract and its bioactive compound EPMC exhibited moderate cytotoxic activity against human CCA tumor (CL-6) cell line. Results of toxicity testing suggest that the extract was well tolerated up to the maximum single oral dose of 5000 mg/kg body weight and daily dose of 1000 mg/kg body weight for 30 days. The extract exhibited promising anti-CCA activity in CL6-xenografed nude mice as determined by significant inhibitory activity on tumor growth and lung metastasis, as well as prolongation of survival time.

## Background

Cholangiocarcinoma (CCA) is one of the important public health problems in Southeast Asia, particularly Thailand. It is an uncommon adenocarcinoma which arises from the epithelial cells of bile ducts anywhere along the intrahepatic and extra hepatic biliary tree excluding the papilla of Vater and the gall bladder [1]. The highest prevalence of CCA in Northeast of Thailand (with age-standardized incidence rate of 33.4 *per* 100,000 in males and 12.3 *per* 100,000 in females) has been associated to the consumption of improperly cooked and preserved cyprinoid fish species which contains the liver fluke, *Opisthorchis viverrini* [2–5]. In other Asian countries like China*,* Korea and Japan, *Clonorchis sinensis* is the main risk factor for CCA [6].

The major challenge for CCA control and treatment is the lack of early diagnosis and resistance of this type of cancer to most chemotherapeutics as well as radiotherapy [7]. At present, surgical resection of detectable tumors and adjunctive therapy with chemotherapeutic agents including gemcitabine, *cis-*platin and *5*- fluorouracil (5-FU) leads to an improvement in the 5-year survival rate, despite low clinical response rate and extremely high recurrence rate [7]. Discovery and development of effective alternative chemotherapeutics for CCA is therefore the first priority needed to be focused.

Plants have formed the basis of traditional medicine systems which have been used for thousands of years and the use of plant-based systems continues to play an essential role in health care. It is estimated that approximately 80% of the population in developing countries rely on traditional medicines for their primary health care [8, 9]. In China, traditional herbal preparations account for 30 to 50% of the medicines consumed [10]. In industrialized countries on the other hand, adaptation of traditional medicine often termed complementary or alternative medicine, also play an important role in the health care system of about 20% of the population [10]. Plants afford a rich repository of remedies with diverse chemical structures and bioactivities against several health disorders including cancers. Several modern anticancer drugs*,* i.e.*,* vinblastine, vincristine, etoposide, teniposide, paclitaxel, vinorelbine, docetaxel, topotecan and irinotecan have been developed from plant sources [11, 12]. Thailand is a country which is rich in a wide range of tropical habitats and remarkable biodiversity. Traditional medicines are used for treatment of various infections and chronic diseases including cancers [13]. Candidate medicinal plants or herbal formulations commonly used in Thai traditional medicine were screened for their anti-CCA activities [11, 14, 15]. Among these, ethanolic extract of the leaves of *Kaempferia galanga* Linn. was shown to exhibit promising in vitro cytotoxic activity against CCA [14]. The highly aromatic rhizome of this plant is valued in Southeast Asian countries as a spice to flavor rice and also in folk medicine. Indigenous medicinal practitioners use the rhizome extract of *Kaempferia galanga* Linn. for various medical purposes including treatment of scariasis, bacterial infections, cancers, cardiotonic and CNS stimulant. In addition, it is also applied externally for abdominal pain in women and for rheumatism [16–18]. Nevertheless, there has been little evidence or report on the in vivo toxicity and anti-CCA activity of this plant*.* The objective of the study was to confirm the anti-CCA potential as well as toxicity of the crude ethanolic extract of *K. galangal* Linn. (rhizome) both in vitro and in animal models.

## Methods

### Chemicals and reagents

Ethanol (95%) used for extraction of *Kaempferia galanga* Linn. rhizome was obtained from Labscan Asia Co. Ltd. (Bangkok, Thailand). HPLC grade methanol and distilled water were purchased from Fisher Scientific (Leicestershire, UK). The cell culture reagents including RPMI 1640, DMEM, fetal bovine serum (FBS), phosphate buffer saline (PBS), penicillin and streptomycin antibiotics were purchased from Gibco Life Technologies (NY, USA). The cholangiocarcinoma cell line (CL-6) was kindly provided by Associate Professor Adisak Wongkajornsilp, Department of Pharmacology, Faculty of Medicine, Siriraj Hospital, Bangkok, Thailand. Methyl thiazoldiphenyl tetrazolium (MTT) was obtained from Life Technologies (CA, USA) and dimethyl sulfoxide (DMSO) was purchased from MP Biomedicals (CA, USA). 5-Fluorouracil (5-FU) was purchased from Wako Pure Chemical Industries Ltd. (Osaka, Japan). Tween-80 was purchased from Sigma-Aldrich (MO, USA). Neutral buffered formalin (NBF, 10%) was purchased from Bio-Optica (Milano, Italy). Isoflurane for euthanasia was purchased from Minrad Inc. (PA, USA). All other chemicals and reagents were high purity grade obtained from commercial suppliers. The standard ethyl-p-methoxy cinnamate (EPMC) was kindly supplied by Dr. Sumet Kongkiatpaibo, Drug Discovery and Development Center, Thammasat University, Thailand**.**

### Preparation of plant extract

The dried and powdered rhizomes of *K. galanga* Linn. was obtained from Nakhon Pathom Province, Thailand. Authentication of plant materials was carried out at the herbarium of the Department of Forestry, Bangkok, Thailand, where the herbarium vouchers have been kept. Preparation of the ethanolic extracts of the plant materials was according to the previously described method [19].

The extract was standardized for extraction efficiency and quality control using high performance liquid chromatography (HPLC) to determine the amount of the marker compound ethyl-p-methoxycinnamate (EPMC) [18]. The HPLC system consisted of HPLC 1200 Series (Agilent Technologies, CA, USA), Hypersil Gold Column (250 × 4.6 mm ID, 5 μm particle size, reversed phase C_18_: Thermoscientific, MA, USA); UV-detector (270 nm, Thermoscientific, MA, USA), and an isocratic solvent of methanol and distilled water (54:46% *v*/v) running at a flow rate of 1 ml/min. The injection volume was 10 μl. The plant extract and standard EMPC were prepared as stock solutions of 20 and 10 μg/ml, respectively.

### In vitro cytotoxic activity of *K. galanga* Linn. Extract and ethyl-p-methoxycinnamate (EPMC)

Cytotoxic activity of the crude ethanolic extract of *K. galanga* Linn. and its bioactive compound ethyl-p-methoxycinnamate (EPMC) against the CCA cell line CL-6 was evaluated according to the previously described method based on the MTT colorimetric assay [20]. The extract, EPMC and the standard control 5-FU were initially dissolved in 50% ethanol to prepare stock solutions (500 μg/ml). Working solutions were prepared at eight final concentrations (500, 250, 125, 62.5, 31.25, 15.6, 7.8, and 3.9 μg/ml) by diluting stock solutions with RPMI 1640 medium. The CL-6 cell line was cultured in T_75_ cm^3^ culture flasks (Corning Inc., NY, USA) with RPMI 1640 medium supplemented with 10% heated fetal bovine serum (FBS) and 100 IU/ml of penicillin-streptomycin solution. The culture was maintained at 37 °C in a 5% CO_2_ atmosphere with 95% humidity. Cultured cells were seeded in 96-well plates (Corning Inc., USA) at a density of 10^4^ cells/well in 100 μl culture medium. Following 24-h incubation and attachment, the cells were treated with various concentrations of the extract, EPMC and 5-FU for 24 h. Following incubation with MTT solution (20 μl of 5 mg/ml in PBS) at 37 °C for 3 h, the media was removed and cells were lyzed with dimethyl sulfoxide (DMSO). Absorbance (OD) was measured at 570 nm using a Varioscan™ flash microplate reader machine (Thermoscientific, MA, USA). Results were evaluated from three independent experiments, triplicate for each experiment. The IC_50_ value (concentration that inhibits cell growth by 50%) was calculated using Calcusyn^R^version 1.1 software (Biosoft, Cambridge, UK). Similarly, cytotoxic activities of the extract, EPMC and 5-FU against normal human fibroblast cell lines (OUMS, cultured in Dulbecco's modified eagle's medium) were evaluated for estimation of selectivity index which is the IC_50_ ratio of the extract or 5-FU in normal cell line (OUMS) and that in cancer cell line (CL-6) [14].

### In vivo toxicity and anti-cholangiocarcinoma activity of *K. galanga* Linn. Extract

#### Animals

The in vivo toxicity of *K. galanga* Linn. extract was evaluated in ICR (Imprinting Control Region) male and female mice, aged 6–7 weeks, and weighing 26–40 g. The anti-CCA activity of the extract was evaluated in BALB/c nude mice models of both genders, aged 7–8 weeks, and weighing 15–24 g. All animal stocks were obtained from the National Laboratory Animal Center, Thailand. Animals were housed under standard conditions and acclimatized for about 1 week before the experiment. The nude mice were maintained in the laboratory in sterilized and individual ventilated cages fitted with molded filter (polyester fiber) covers and all experimental procedures were conducted in a biosafety cabinet to maintain specific pathogen free condition. Mice were allowed free access to food (standard pellet) and water ad libitum throughout the experimental period. The temperature of the room was maintained at 20-21 °C with relative humidity of 47–53%. Good hygiene was maintained by constant cleaning and removal of feces and spilled feed from cages twice a week. Effort was made to minimize animal suffering during the experimental period. The animals were handled according to the International Guidelines for Animal Welfare and the study protocol was approved by the Ethics Committee for Animal Research of Thammasat University, Thailand (Ethical approval number 005/2558).

#### In vivo toxicity (acute and subacute) evaluation

Acute and subacute toxicity tests were performed in ICR mice according to the OECD (International Organization for Economic Co-operation and Development) Guideline for Chemicals [21, 22] with modifications, to obtain the three dose levels (maximum tolerated, medium, and low dose levels) of each test material which did not cause any unacceptable sign of toxicity or death. These three tolerated dose levels were further used for assessment of the anti-CCA and pharmacological activities of each test material. The mice (5 males and 5 females for each group) were fed (via gastric gavage) with three dose levels (starting from the highest dose of 5000 mg/kg bodyweight and lowered down to the two additional dose levels, i.e., medium and low doses) of the test materials (resuspended in a mixture of distilled water and Tween-80 4:1, v:v). The control animals were fed with a mixture of distilled water and Tween-80. Animals were closely observed for signs of toxicity during the first 30 min, periodically during the first 24 h, and then daily for 14 days (acute toxicity) or 30 days (subacute toxicity).

At the end of the observational period (on the 15th day), all animals were sacrificed by euthanasia with isoflurane anaesthesia followed by cervical dislocation. Vital organs (brain, heart, kidneys, liver, spleen, stomach, large and small intestine, and lungs) were removed from all animals for gross and histopathological examination. Blood samples were collected in vacutainer tubes (with and without EDTA anticoagulant) at terminal stage following euthanasia of mice for estimation of hematology and serum chemistry parameters. Histopathological tissue processing and specimen staining with hematoxylin and eosin was performed on the preserved organs according to the previously described methods [15, 23]. The morphological changes within the tissues of the control and treated groups of mice were observed under binocular compound light microscope with camera (Leica Microsystems, Wetzlar, Germany) at 100× (oil immersion) magnification.

### Anti-cholangiocarcinoma activity in CCA-xenografted nude mice

The CCA cell line CL-6 (1 × 10^6^ cells) was used for xenografting all nude mice. Cells were removed from culture flask by trypsinization, collected in a 50 ml conical tube and centrifuged at 100×g for 10 min. Supernatant was removed and cells were resuspended in 5 ml of complete media and cell number was counted using hemocytochamber. Cells for injection were prepared by diluting cell suspension to obtain 1 × 10^6^ cells/200 μl normal saline (0.85% NaCl) and injected subcutaneously into the right upper flanks of the nude mice [24]. Tumors were allowed to develop for 14 days until they reached approximately 50 mm^3^ tumor volume. Tumor volume was measured using a caliper. Body weight was recorded once every three days.

To evaluate the anti-CCA activity of the extract, CCA-xenografted nude mice were divided into groups of 6 mice (3 males and 3 females) for each dose level, matched-pair according to tumor size (approximately to 50 mm^3^ tumor volume), and body weight. The extract was given at the three dose levels, i.e.*,* high (maximum tolerated dose of 1000 mg/kg body weight, medium dose of 500 mg/kg body weight, and low dose of 100 mg/kg body weight) based on the maximum tolerated dose observed in the toxicity test. The extract was fed to animals by intragastric tube (0.3 ml) daily for 30 days. The untreated and 5-FU treated control groups were fed with an equal volume of normal saline daily for 30 doses and 40 μg/ml for 14 doses, respectively. On day 0 (the day of first dose), 2, 5, 8, 11, 14, 18, 21, 24 and 28, animals were weighed on a triple-beam balance and tumor size was measured in two linear dimensions (maximum longitudinal and transverse diameters) using a caliper with accuracy of 0.1 mm. The tumor volume (TV) was calculated from the formula: tumor volume = (length) (width)^2^/2 [25]. Tumor growth inhibition (TGI) was calculated at the end of treatment period (on day 31th) as follows: TGI (%) = (TV control-TV treatment) ×100/TV control. For survival time determination, date of death of each mouse was recorded following treatment initiation. Mice were sacrificed with CO_2_ euthanasia [26] when the growing tumor burden impaired their locomotion and other activities as well as ability to eat or drink (protocol end points). The median survival time (in days) of CCA-xenografted nude mice receiving test extract and reference control at all dose levels after initiation of treatment were compared with the untreated (vehicle) group. Tumor metastasis to other organs (lungs, kidneys, heart, liver, and spleen) was examined macro- and microscopically.

#### Statistical analysis

Statistical analysis was performed using SPSS software version 18. Qualitative variables are presented as number (n) and/or percent (%). Quantitative data are presented as median and 95% confidence interval (95%CI). Comparison of quantitative variables between two or more than two groups was performed using Mann-Whitney U-test and Kruskal Wallis test (followed by pairwise comparison), respectively. Statistical significance level was set at α = 0.05 for all tests.

## Results

### *K. galanga* Linn. Rhizome extract and marker identification

**Fig. 1:**
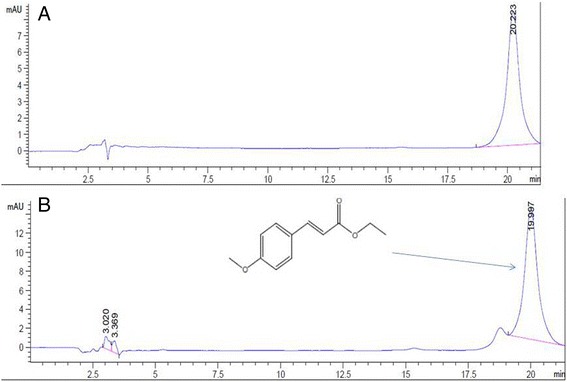
HPLC chromatograms of **a** standard ethyl-*p*-methoxycinnamate (EPMC) and **b** *K. galangal* Linn.rhizome extract. Chromatographic separation condition used was as follows: Thermoscientific^™^ Hypersil Gold Column 5 μm C18 column; mobile phase consisting of a mixture of water and methanol with isocratic elution (46%: 54% *v*/v) at follow rate of 1 ml/min; injection volume of 10 μl; and UV-detection at 270 nm

The ethanolic extract of *K. galanga* Linn. rhizome was a semi-solid oily solution, with fruity odor and amber color**.** The extract was evaluated for content of the biological active marker compound ethyl-p-methoxycinnamate (EPMC). HPLC analysis of the extract showed a peak at the same retention time of EPMC, i.e.*,* 19.99 min, with peak area of 94.09% of the total peak area (Fig. 1).

### In vitro cytotoxic activity of *K. galanga* Linn. Rhizome extract and EPMC

*K. galanga* Linn. extract exhibited 80% and 94% inhibitory activity on CL-6 cell growth at 125 and 250 μg/ml, respectively. The median (95%CI) IC_50_ values of the extract, EPMC and 5-FU were 64.2 (57.76–72.11), 49.19 (48.16–52.29) and 107.1 (103.53–109.64) μg/ml, respectively. The corresponding IC_50_ values of the extract, EPMC and 5-FU against OUMS cell line were 138.79 (138.43–160.5), 103.18 (99.17–110.84) and 140.38 (132.11–146.04) μg/ml, respectively. The selectivity index (SI) values for the extract, EPMC and 5-FU were 2.2, 2.09 and 1.31, respectively.

### In vivo toxicity of *K. galanga* Linn. Rhizome extract

#### Acute toxicity test

Mice receiving *K. galanga* Linn. rhizome extract and untreated control (20% tween-80) showed no sign of toxicity (general behavior, body weight change, and histopathology of vital organs) at a single highest dose level of 5000 mg/kg body weight, with no death during the observation period of 14 days.

#### Subacute toxicity test

Three (30%) and 4 (40%) mice receiving 5000 mg/kg (highest dose level) and 3000 (medium dose level) of the extract respectively, died. All of the deaths were observed during the first two weeks of dosing. All mice receiving the lowest dose level of 1000 mg/kg body weight of the extract and the control mice receiving 20% Tween-80 survived during the 30 day observation period. The maximum tolerated dose level of the extract was therefore 1000 mg/kg. The animals did not show any major changes in general behavior or other physiological activities (including respiratory pattern, cardiovascular signs, motor activities, reflexes, feeding and drinking activities) particularly at the low dose level and in the control group. At 5000 and 3000 mg/kg body weight dose levels however, transient signs of reduced activity (lethargy) and piloerection were observed in both male and female mice. No remarkable histopathological change was detected in cellular morphology and general architecture of tissues performed on the internal organs between different dose levels and control. Minor forms of microvascular haemorrhage in some organs, i.e.*,* liver, lungs and heart were observed in both treated and control groups of mice, which was likely to be associated to bleedings occurred during autopsy and trimming process of the respective tissues for specimen collection.

All of the hematological and biochemical parameters of all mice were within normal ranges [27]. Significant changes in some of the parameters were observed in mice receiving the extract compared with control mice. Since data were available only for the dose level of 1000 mg/kg body weight in the group of female mice, comparison with control was possible only for this dose level. In male mice, a trend of increase in hematocrit value was observed at all dose levels of the extract, but significant difference was only found with the extract at the medium dose level of 3000 mg/kg body weight. The mean corpuscular hemoglobin concentration (MCHC) was significantly decreased at all dose levels compared with control (Table 1a). For serum biochemistry, a trend of increase in BUN levels was observed in male mice receiving all dose levels, but significant difference from the control group was only detected with the high dose of 5000 mg/kg body weight (Table 2a). Furthermore, a significant increase in the AST level was observed in female mice receiving 1000 mg/kg body weight of the extract (Table 2b).

**Table 1:** Haematological profiles of (A) male and (B) female mice at the end of subacute toxicity dosing of *K. galanga* Linn. rhizome extract (1000, 3000, and 5000 mg/kg body weight) and untreated control mice. Data are presented as median (95% CI)

Control		*K. galanga* Linn. rhizome extract (mg/kg body weight)		
		1000	3000	5000
A				
Red blood cells (×10^6^/μl)	7.31 (6.16–8.46)	8.13 (6.90–9.36)	8.11 (7.59–8.62)	7.92 (7.49–8.35)
Hemoglobin (g/dl)	12.27 (9.83–14.71)	12.80 (11.48–14.11)	13.03 (12.38–13.67)	12.43 (12.03–12.82)
Hematocrit (%)	34.7 (29.5–39.8)	42.0 (37.7–46.3)	43.5 (37.5–49.5)*	41.8 (40.9–42.6)
MCV	47.1 (46.19–47.94)	51.17 (46.48–55.86)	53.85 (45.19–62.51)	52.80 (50.46–55.13)
MCH (pg)	16.8 (15.73–17.80)	15.77 (14.00–17.53)	16.10 (14.69–17.51)	15.73 (14.99–16.45)
MCHC (g/dl)	35.6 (32.89–38.37)	30.8 (30.21–31.46)*	30.0 (27.48–32.52)*	29.8 (28.66–30.84)*
White blood cells (×10^3^/μl)	5.30 (1.02–9.58)	3.91 (3.30–4.53)	5.38 (3.98–6.79)	3.63 (2.90–4.35)
Platelets (×10^5^/μl)	6.29 (1.73–10.85)	10.67 (7.08–14.27)	7.43 (2.94–11.92)	6.53 (1.03–13.02)
B				
Red blood cells (×10^6^/l)	7.59 (4.95–10.22)	6.92 (3.78–13.88)	NA	NA
Hemoglobin (g/dl)	13.44 (11.89–14.99)	12.60 (8.85–16.35)	NA	NA
Hematocrit (%)	37.80 (24.50–51.10)	34.00 (20.47–54.53)	NA	NA
MCV	49.82 (48.83–50.82)	49.43 (40.10–58.76)	NA	NA
MCH (pg)	19.06 (11.39–26.73)	18.20 (7.01–29.39)	NA	NA
MCHC (g/dl)	38.22 (23.21–53.23)	36.53 (13.32–59.75)	NA	NA
White blood cells (×10^3^/μl)	3.66 (1.03–6.28)	4.15 (2.78–5.53)	NA	NA
Platelets (×10^5^/μl)	4.36 (0.91–7.81)	3.03 (0.44–6.05)	NA	NA

^*^Significantly different from the control group, *p* = 0.01, 0.23, and 0.21 for the dose levels of 1000, 3000 and 5000 mg/kg body weight, respectively NA, data not available due to death or small sample. No statistical significant difference (*p* > 0.05) for the parameters evaluated between the treatment groups and control group

**Table 2:** Serum biochemistry profiles of (A) male and (B) female mice at the end of subacute toxicity dosing of K. galanga Linn. rhizome extract (1000, 3000, and 5000 mg/kg body weight) and untreated control mice. Data are presented as median (95% CI)

Control		*K. galanga* Linn. rhizome extract (mg/kg body weight)		
		1000	3000	5000
A				
BUN (mg/dl)	18.7 (15.8–21.54)	22.0 (15.4–28.6)	23.5 (19.71–27.29)	28.3 (21.09–35.41)*
Creatinine (mg/dl)	0.13 (0.10–0.27)	0.23 (0.14–0.61)	0.20 (0.20–0.20)	0.20 (0.07–0.33)
Total protein (g/dl)	4.70 (4.45–4.95)	4.77 (4.48–5.05)	5.62 (4.37–6.88)	5.05 (4.75–5.36)
Albumin (g/dl))	3.03 (2.89–3.18)	2.90 (2.65–3.15)	3.15 (2.46–3.84)	3.00 (2.78–3.23)
AST (U/l)	57.0 (27.2–86.8)	97.0 (20.5–173.5)	109.5 (26.7–192.3)	87.8 (59.7–115.8)
ALT (U/l)	39.0 (21.6–56.4)	53.3 (27.9–134.6)	68.5 (11.5–148.5)	56.5 (38.3–74.7)
ALP (U/l)	90.0 (85.0–95.0)	79.0 (57.5–101)	68.5 (51.5–85.5)	77.3 (54.4–100.1)
Sodium (mEq/L)	150.3 (144.1–156.6)	150.3 (140.9–159.7)	149.5 (143.3–155.7)	150.5 (146.5–154.5)
Potassium (mEq/L)	11.5 (9.9–13.2)	10.7 (5.6–15.9)	13.7 (8.9–18.5)	12.9 (10.8–15.0)
Cholesterol (mg/dl)	113.0 (100.6–125.4)	109.0 (95.17–122.8)	153.7 (69.27–238.2)	126.3 (108.2–144.3)
Triglycerides (mg/dl)	81.00 (31.32–130.7)	135.3 (83.15–187.5)	242.3 (68.70–415.8)	207.0 (147.9–266.0)
HDL (g/dl)	113.0 (110.5–115.5)	99.67 (83.70–115.6)	137.8 (67.01–208.5)	125.0 (94.48–155.5)
LDL (mg/dl)	12.0 (9.5–14.5)	9.7 (4.5–14.8)	13.5 (4.3–22.7)	9.0 (4.7–13.3)
B				
BUN (mg/dl)	19.25 (15.07–23.43)	22.33 (20.9–23.77)	NA	NA
Creatinine (mg/dl)	0.15 (0.06–0.24)	0.13 (0.01–0.27)	NA	NA
Total protein (g/dl)	4.55 (4.27–4.83)	4.63 (4.01–5.26)	NA	NA
Albumin (g/dl))	3.13 (3.05–3.21)	3.20 (2.95–3.45)	NA	NA
AST (U/l)	56.75 (49.83–63.67)	92.33 (78.65–106.01)*	NA	NA
ALT (U/l)	23.25 (16.71–29.79)	23.67 (2.54–44.79)	NA	NA
ALP (U/l)	101.75 (83.78–119.72)	97.33 (86.13–108.53)	NA	NA
Sodium (mEq/L)	150.75 (148.03–153.47)	151.03 (151.00–151.06)	NA	NA
Potassium (mEq/L)	8.45 (6.14–10.77)	8.40 (7.90–8.90)	NA	NA
Cholesterol (mg/dl)	66.75 (51.58–81.92)	69.67 (18.74–120.60)	NA	NA
Triglycerides (mg/dl)	94.50 (60.96–120.04)	117.33 (88.46–136.21)	NA	NA
HDL (g/dl)	67.00 (53.56–80.44)	63.00 (18.29–107.71)	NA	NA
LDL (mg/dl)	8.50 (5.19–11.81)	10.00 (2.55–17.45)	NA	NA

^*^Significantly different from the control group, *p* = 0.01 NA, data not available due to death or small sample ^*^Significantly different from the control group, *p* = 0.012

### Anti-cholangiocarcinoma activity of *K. galanga* Linn. Extract against CCA-xenografted nude mice

**Fig. 2:**
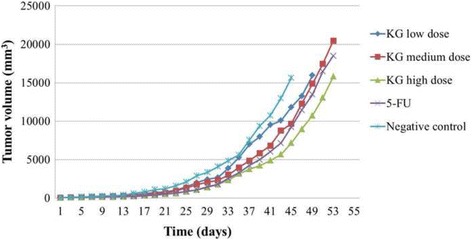
Anti-CCA activity and tumor volume (TV) progression (mm^3^) in CCA (CL6)-xenografted nude mice following initiation of treatment with *K. galanga* Linn. extract at high (1000 mg/kg body weight), medium (500 mg/kg body weight), and low (100 mg/kg body weight) dose levels versus the control groups (vehicle treated and 5-FU treated) during the investigation period. Treatment was started on day 7th after tumor transplant induction. Median survival time of the CCA-xenografted nude mice treated with the high dose extract [1000 mg/kg body weight: 62 (53.2–71.8) days] and 5-FU [59.0 (55.0–63.0) days] were significantly longer than the untreated control mice [49 (54.4–52.6) days]. Each data point is the median value of 6 tumors (from 3 male and 3 female mice) for each treatment group

**Fig. 3:**
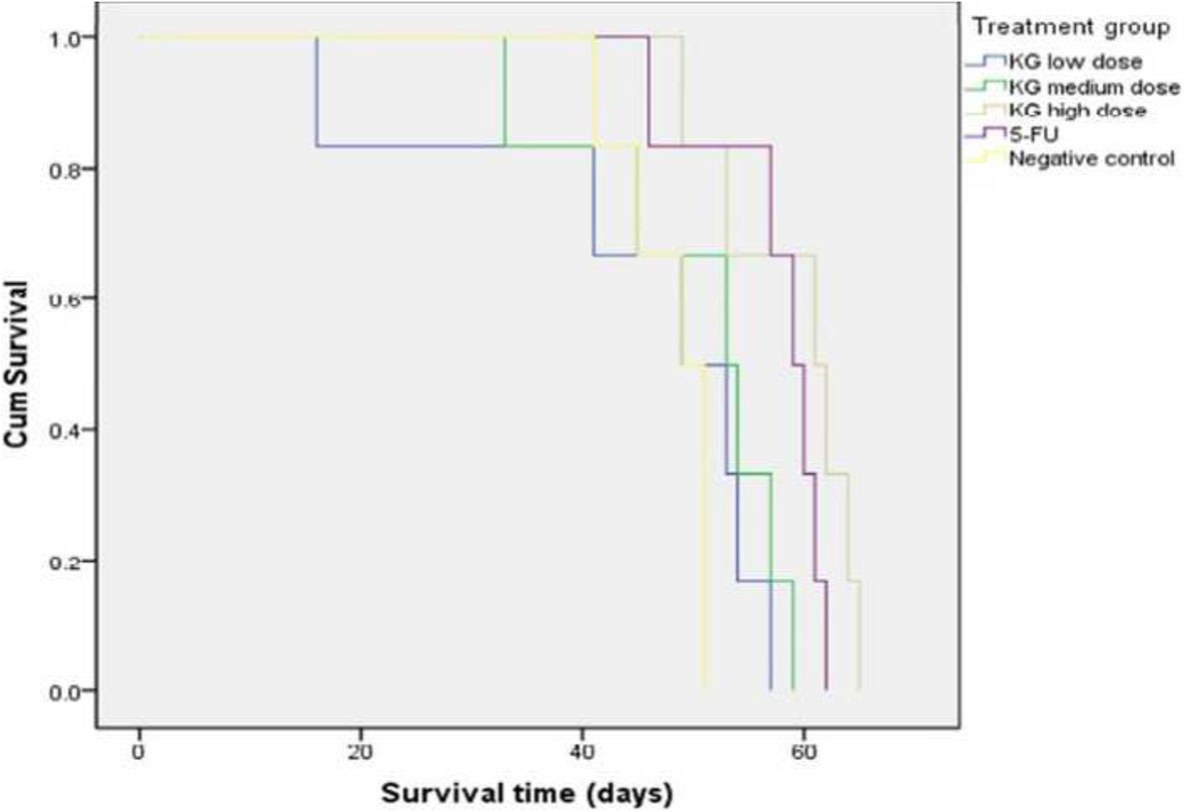
Median survival time (days) of CCA-xenografted nude mice receiving the three dose levels of *K. galanga* Linn, i.e., high (1000 mg/kg body weight), medium (500 mg/kg body weight) and low (100 mg/kg body weight), and reference drug (5-FU) during the observation period (3 male and 3 female mice for each group). Abbreviations: KG = *K. galanga*l Linn., 5-FU = 5-fluorouracil

The median (95% CI) TV at the end of treatment period (on day 31th) for the groups treated with the extract at high (1000 mg/kg body weight), medium (500 mg/kg body weight) and low (100 mg/kg body weight) dose levels were 1390 (933–1846), 2067 (1074–3059), and 2438 (1421–3455) mm^3^, respectively. The TV for 5-FU treated and the untreated control mice were 1441 (929–1953) and 3342 (2578–4105) mm^3^, respectively (Fig. 2). The corresponding TGI in mice receiving the extract at 1000, 500 and 100 mg/kg body weight, 5-FU and untreated control were 58.41, 38.15, 27.04, 56.87 and 0% of control, respectively (Table 3). The median survival time of the CCA-xenografted nude mice treated with the high dose extract (1000 mg/kg body weight) and 5-FU [62 (53.2–71.8) and 59.0 (55.0–63.0) days] were significantly longer (*p* = 0.01) than the untreated control mice [49 (45.4–52.6) days]. The survival time of mice receiving medium and low dose extract were 53 (42.2–63.8) 49 and (34.6–63.4) days, respectively (Fig. 3).

**Table 3:** Representative tumor metastasis and proportion of mice with metastasis in CL-6 xenografted nude mice treated with *K. galanga* Linn. extract and control (untreated and 5-FU) groups

Treatment group	Time at autopsy (weeks)	Lung metastasis	
		Macrometastasis	Proportion of mice without metastasis
Low dose *K. galanga* Linn. extract (100 mg/kg body weight)	3–9		0/6 (0%)
Medium dose *K. galanga* Linn. extract (500 mg/kg body weight)	5–9		1/6 (16.7%)
High dose *K. galanga* Linn. extract (1000 mg/kg body weight)	7–10		2/6 (33.3%)
Untreated control	6–7		0/6 (0%)
5-FU treated (40 mg/kg body weight)	7–9		1/6 (16.7%)

At autopsy, mice were examined visually for primary tumor observation and distant metastases. Macro-metastases were found in all the lungs of control and mice receiving 1000 mg/kg body weight extract. The proportions of mice receiving 1000, 500, and 100 mg/kg body weight extract and 5-FU without lung metastases were 33.3 (2/6), 16.7% (1/6), 0% (0/6) and 16.7% (1/6), respectively (Table 3).

## Discussion

Qualitative analysis of the marker EPMC in the test extract was made between sample retention time with retention times of the standard. From the high content of volatile oil in the rhizome of *K. galangal* Linn., the marker compound EPMC was detected using HPLC with a major peak area of about 94.09%. As one of the phenolic compounds, the chemical marker EPMC, in *K. galangal* can be considered to have higher solubility in organic solvents including dichloromethane and ethanol as compared with that of water [28]. Apart from CCA, this compound has been shown to inhibit proliferation of human hepatocellular liver carcinoma (Hep G2 cell line) in a dose-dependent manner and annexin-fluorescein isothiocyanate and propidium iodide staining showed an increased early apoptotic population in human hepatocellular carcinoma cells [29]. Variation of the content of EPMC from the volatile oil of *K. galanga* Linn. was reported from various studies as 31.77% [18] and 80% [30]. This variation could be due to different extraction and analytical procedures. In addition, phytochemicals are mostly minor plant constituents whose concentration varies considerably according to seasonal and agronomic factors, the variety, age, and part of the plant examined [31].

Results of cytotoxicity test showed moderate activity of both the *K. galanga* Linn. rhizome extract and its bioactive compound EPMC against CL-6 cell lines with median IC_50_ of 64.2 and 49.9 μg/ml, respectively. Their potency of cytotoxicity and selectivity on CCA cells was similar, but was about 1.5 to 2-fold of 5-FU (107.1 μg/ml). It was noted however for the relatively low potency of the extract (about 50%) compared with that reported during the initial screening of the leaf extract against the same CL6 cell line (mean ± SD IC_50_ = 37.36 ± 3.98 μg/ml, SI = 2.9) [14]. The IC_50_ of the plant extract and 5-FU appears to be too high above recommended threshold for any compound or extract to be regarded as anticancer agent. CCA is the cancer that is highly resistant to anticancer drugs and this multidrug resistant nature might explain the low sensitivity of this type of cancer to chemotherapeutics including those obtained from medicinal plant sources [4, 32]. There is a general agreement between a particular tumor type and its corresponding clinical cancer with respect to their response to a given drug or set of drugs [33]. Since the tumor cell line (CL-6)was derived from human CCA tumor, the patient from whom this tumor line was obtained might have received 5-FU based chemotherapy. This may explain the relatively low sensitivity of the cells to 5-FU compared with the extract. With regard to cytotoxic activity against other cancer cells, the ethanolic extract of *K. galanga* Linn. rhizomes has been reported to exhibit potent activity against SW 620 (human colorectal adenocarcinoma cell line (IC_50_ = 6.13 ± 0.52 μg/ml), DU145 (human prostate cancer cell line (IC_50_ = 10.51 ± 0.34 μg/ml), PA1 (human ovarian teratocarcinoma cell line (IC_50_ = 10.53 ± 0.22 μg/ml), and B16F10 (murine melanoma cell line (IC_50_ = 12.63 ± 1.24 μg/ml) [34]. The discrepancy in cytotoxic activities of the extract against various cancer cells could be associated with variations in geographic origins of the herb or extraction methods, and in particular, difference in sensitivity of the cancer cells.

Results from the acute toxicity test with *K. galangal* Linn. extract indicated virtually no toxicity with respect to mortality and morbidity (body weight changes, internal organ weights, and signs of abnormalities of the internal organs at gross and microscopic levels) at the highest dose level of 5000 mg/kg body weight (single oral dose). In the subacute toxicity test (daily doses for 30 days), relatively low toxicity was observed up to the dose of 1000 mg/kg body weight. At higher dose levels (3000 and 5000 mg/kg body weight), deaths of mice and behavioral signs of lethargy and piloerection were observed. Nevertheless, significant differences in body weight changes, organ weights, post-mortem gross organ lesions and histopathological changes were not observed between different dose levels and control. The effect of the extract on mortality of mice was dose-independent manner and only observed during the first two weeks. Comparatively higher frequency of death incidence was observed in mice receiving the extract at the dose of 3000 compared with 5000 mg/kg body weight (4/10 vs. 3/10 mice). This difference was minor and could be explained by variability in the response of the animals as well as the complication of the gavage methodology [35]. The observation of death during the first two weeks of the subacute toxicity period suggests immediate toxic effect of the extract which could lead to severe adverse effects such as organ function loss leading to death [36]. In a study conducted in mice with the dichloromethane extract of *K. galanga* Linn. relatively lower dose level (100 mg/kg body weight) and EPMC (120 and 160 mg/kg body weight) for 28 consecutive days, no significant signs of morbidity was observed [30]. In rats, no toxicity was observed when treated with the extract at the oral dose of up to 100 mg/kg body weight [37].

With regard to the toxicity of the extract on hematological and biochemical profiles, most of values except hematocrit, MCHC, BUN and AST, were not significantly different between the extract treated and control groups. This was in agreement with that previously reported in rats in a subacute toxicity test of *K. galanga* Linn. rhizome extract at the oral dose of up to 1000 mg/kg body weight [38]. A significant increase in hematocrit (3000 mg/kg body weight) and a decrease in MCHC (all the 3 dose levels) were observed in male mice compared with control. In addition, a significant increase in BUN and AST was found in male and female mice receiving the extract at the dose levels of 5000 and 1000 mg/kg body weight, respectively. The observed variability in toxicity in male and female mice could be due to normal variation among animal groups. The changes in some of the laboratory parameters however, remained within the normal ranges reported in mice, suggesting that such variations were not associated with the *K. galangal* extract [27, 38].

The results from the current toxicity study indicate that oral administration of *K. galanga* Linn. extract up to 1000 mg/kg body weight was well tolerated and this dose level was therefore considered as the maximum tolerated dose level for further evaluation of anti-CCA activity of the extract in CCA-xenografted nude mouse model. The nude mouse/human tumor xenograft system provides a useful model for cancer therapy studies involving human neoplasms. Moreover, the system lends itself to the development of screening protocols for the identification of potential anticancer drugs which would be clinically effective against a given type of cancer [24, 33]. Despite the fact that not all human tumors can be successfully xenografted, the histology and biochemical properties of the tumors that do grow in nude mice closely resemble those of the original tumor specimens [25]. The rhizome extract of *K. galanga* Linn. at 1000 (high) and 500 (medium) mg/kg body weight and 5-FU showed significant anti-CCA activity in CL6-xenografted nude mice based on TV progression, TGI and inhibitory on lung metastasis compared with the control group. The anti-CCA activity of the extract was clearly seen at the high dose level of 1000 mg/kg body weight (TGI 58.41%, median survival time 62 days, proportion of mice without lung metastasis 33.3%). Although the extract and 5-FU did not arrest tumor growth or progression during the observation period (Fig. 3), the rate of tumor growth was considerably slow particularly for the high dose extract and 5-FU treated groups. The rapid increase in TV progression observed in all groups might be due to multidrug resistance nature of the CCA tumor [32]. A significant prolongation of the mean survival time of CCA-xenografted nude mice treated with 5-FU compared with untreated control (55 ± 0.87 days vs 40.0 ± 0.57 days) was also reported in our previous study [15]. Metastasis is a major cause of treatment failures and death in many cancers including CCA. Macrometastasis examination at autopsy in this study revealed lung metastasis of CL-6 tumor in all mice receiving low dose extract and untreated control mice in the current study. The high metastatic rates observed in these groups could be associated with the higher respective tumor burdens in these groups. In addition, delay in the autopsy time due to prolongation of the survival time observed in most animals led to metastatic spread of the tumor to the lungs.

## Conclusions

Results of the present and previous studies suggest that the *K. galangal* Linn. rhizome extract and its bioactive compound EPMC exhibited moderate cytotoxic activity against human CCA tumor (CL-6) cell line. Although result of cytotoxicity test suggests relatively low selectivity of the extract on CCA cells, results of toxicity testing revealed no overt toxicity up to the maximum single oral dose of 5000 mg/kg body weight and daily dose of 1000 mg/kg body weight for 30 days. The extract at the maximum tolerated dose of 1000 mg/kg body weight for 30 days exhibited promising anti-CCA activity in CL6-xenografed nude mice as determined by significant inhibitory activity on tumor growth and lung metastasis, as well as prolongation of survival time. In an effort to develop an effective alternative treatment option against CCA, further studies should be carried out to confirm its tolerability profile following chronic dosing, as well as pharmacological activities, molecular and cellular mechanisms of action, and pharmacokinetics of the bioactive compound EPMC. In vivo evaluation of anti-CCA activity of EPMC in animals may not be required considering the markedly low concentrations and equipotent cytotoxic activity of both the crude extract and the pure compound EPMC against CCA cells. It is likely that other unidentified constituents in the extract may act synergistically to produce anti-CCA.

## Abbreviations

5-FU: 5-fluorouracil
CCA: Cholangiocarcinoma
EPMC: Ethyl-p-methoxycinnamate
OECD: International Organization for Economic Co-operation and Development
SI: Selectivity index

## References

[1] Mosconia S, Berettab G, Labiancaa R (2009). Cholangiocarcinoma. Crit Rev. Oncol Hematol.

[2] Haswell-Elkins MR, Satarug S, Elkins DB (1992). *Opisthorchis viverrini* Infection in northeast Thailand and its relationship to cholangiocarcinoma. J Gastroenterol Hepatol.

[3] Khuhaprema T, Srivatanakul P (2008). Colon and rectum cancer in Thailand: an overview. Jpn J Clin Oncol.

[4] Dholwani KK, Saluja AK, Gupta AR, Shah DR (2008). A review on plant-derived natural products and their analogs with anti-tumor activity. Indian J Pharmacol.

[5] Singh MK, Facciuto ME (2012). Current management of cholangiocarcinoma. Mt Sinai J Med.

[6] WHO: Cancer Epidemiology. https://www.iarc.fr/en/publications/pdfs-online/epi/index.php (2003). Accessed on 2 Dec 2015.

[7] Ramírez-Merino N, Aix SP, Cortés-Funes H (2013). Chemotherapy for cholangiocarcinoma: an update. World J Gastrointest Oncol.

[8] Hostesttmann K, Marston A (2002). Twenty years of research into medicinal plants: results and perspectives. Phytochem Rev.

[9] Zia-Ul-Haq M, Stanković M, Rizwan K, De Feo V (2013). *Grewia asiatica* L., a food plant with multiple uses. Molecules.

[10] WHO: Traditional medicine. http://www.who.int/mediacentre/factsheets (2003). Accessed on 10 Dec 2015.

[11] Na-Bangchang K, Karbwang J (2014). Traditional herbal medicine for the control of tropical diseases. Trop Med Health.

[12] Kinghorn AD, DE Blanco EJ, Lucas DM, Rakotondraibe HL, Orjala J, Soejarto DD, Oberlies NH, Pearce CJ, Wani MC, Stockwell BR, Burdette JE, Swanson SM, Fuchs JR, Phelps MA, Xu L, Zhang X, Shen YY (2016). Discovery of anticancer agents of diverse natural origin. Anticancer Res.

[13] Subchareon P (1998). Handbook of anticancer: Thai traditional medicine: new concept for treating cancer.

[14] Mahavorasirikul W, Viyanant V, Chaijaroenkul W, Itharat A, Na-Bangchang K (2010). Cytotoxic activity of Thai medicinal plants against human cholangiocarcinoma, laryngeal and hepatocarcinoma cells in vitro. BMC Complement Altern Med.

[15] Plengsuriyakarn T, Viyanant V, Eursitthichai V, Picha P, Kupradinun P, Itharat A, Na-Bangchang K (2012). Anticancer activities against cholangiocarcinoma, toxicity and pharmacological activities of Thai medicinal plants in animal models. BMC Complement Altern Med.

[16] Mokkhasmit M, Swatdimongkol K, Satrawah P (1971). Study on toxicity of Thai medicinal plants. Bull Dept Med Sci.

[17] Hirschhorn H (1983). Botanical remedies of the former Dutch east indies (Indonesia). J Ethnopharmacol.

[18] Tewtrakul S, Uuenyongsawad S, Kummee S, Atsawajaruwan L (2005). Chemical components and biological activities of volatile oil of *Kaempferia galanga* Linn. Songklanakarin J Sci Technol.

[19] Sasidharan S, Chen Y, Saravanan D, Sundram K, Yoga Latha L (2011). Extraction, isolation and characterization of bioactive compounds from plants’ extracts. Afr J Tradit Complement Altern Med..

[20] Mosmann T (1983). Rapid colorimetric assay for cellular growth and survival: application to proliferation and cytotoxicity assays. J Immunol Methods.

[21] OECD. Test No. 420: Acute Oral Toxicity - Fixed Dose Procedure: OECD Publishing; [2001cited 2015] Available from:/content/book-943,070,264,789,9/en. doi:10.1787/9789264070943-en.

[22] OECD. Test No: 407. Repeated Dose-28 day Oral Toxicity Study in Rodents: OECD Publishing [2008 cited 2015 Jan 3] Available from:/content/book/9789264070684-en10.1787/9789264070684-en

[23] Sireeratawong S, Jaijoy K, Panunto W, Nanna U, Lertprasertsuke N, Soonthornchareonnon N (2013). Acute and chronic toxicity studies of the water extract from dried fruits of Terminalia bellerica (Gaertn.) Roxb. In Spargue-Dawley rats. Afr J Tradit Complement Altern Med.

[24] Ovejera AA, Houchens DP (1981). Human tumor xenografts in athymic nude mice as a preclinical screen for anticancer agents. Semin Oncol.

[25] Giovanella BC, Fogh J (1985). The nude mouse in cancer research. Adv Cancer Res.

[26] AVMA (2013). The AVMA guidelines for the euthanasia of animals.

[27] Suckow M, Danneman P, Brayton C (2001). The laboratory mouse.

[28] Funk C, Brodelius P (1992). Phenylpropanoid metabolism in suspension cultures of vanilla planifolla Andr. IV. Induction of vanillic acid formation. Plant Physiol.

[29] Liu B, Liu F, Chen C, Gao H (2010). Supercritical carbon dioxide extraction of ethyl p-methoxycinnamate from *Kaempferia galanga* L. rhizome and its apoptotic induction in human HepG2 cells. Nat Prod Res.

[30] Sirisangtragul W, Sripanidkulchai B (2011). Effects of *Kaempferia galanga* L. and ethyl-p-methoxycinnamate (EPMC) on hepatic microsomal cytochrome P450s enzyme activities in mice. Songklanakarin J Sci Technol.

[31] Birgit H, Gary W: Methods to study bioavailability of Phytochemicals. In: Phytochemicals in health and disease. edn. Edited by Yongping B, Roger F. NYs: MARCEL DEKKER, INC.; 2004: 25-56.

[32] Namwat N, Amimanan P, Loilome W, Jearanaikoon P, Sripa B, Bhudhisawasdi V, Tassaneeyakul W (2008). Characterization of 5-fluorouracil-resistant cholangiocarcinoma cell lines. Chemotherapy.

[33] Ovejera A, Houchens D, Barker A (1978). Chemotherapy of human tumor Xenografts in genetically Athymic mice. Ann Clin Lab Sci.

[34] Jagadish P, Raghu C, Vinod K, Latha K: Potent selective cytotoxic activity of Kaempferia galanga L. rhizome against cancer cell cultures. Int J Pharm Bio Sci. 2010;1(2):105.

[35] Balcombe J, Barnard N, Sandusky C (2004). Laboratory routines cause animal stress. Contemp Top Lab Anim Sci.

[36] Simon F, Tarah P.The Ethics of Research Involving Animals: A Review of the Nuffield Council on Bioethics. Altern Lab Anim. 2005;33(6):654–58. 10.1177/026119290503300603 16419360

[37] Kanjanapothi D, Panthong A, Lertprasertsuke N, Taesotikul T, Rujjanawate C, Kaewpinit D, Sudthayakorn R, Choochote W, Chaithong U, Jitpakdi A (2004). Toxicity of crude rhizome extract of *Kaempferia galanga* L. (Proh Hom). J Ethnopharmacol.

[38] Feldman B, Zinkl J, Jain N, Moor D (2000). Schalm’s veterinary hematology.

